# Developing de novo human artificial chromosomes in embryonic stem cells using HSV-1 amplicon technology

**DOI:** 10.1007/s10577-014-9456-2

**Published:** 2015-02-06

**Authors:** Daniela Moralli, Zoia L. Monaco

**Affiliations:** 1 The Wellcome Trust Centre for Human Genetics, University of Oxford, Roosevelt Drive, Oxford, OX3 7BN UK; 2 Department of Biomedical Engineering, Tufts University, 4 Colby Street, 02155-6013 Medford, MA USA

**Keywords:** gene therapy, gene expression, viral replication, viral vector

## Abstract

De novo artificial chromosomes expressing genes have been generated in human embryonic stem cells (hESc) and are maintained following differentiation into other cell types. Human artificial chromosomes (HAC) are small, functional, extrachromosomal elements, which behave as normal chromosomes in human cells. De novo HAC are generated following delivery of alpha satellite DNA into target cells. HAC are characterized by high levels of mitotic stability and are used as models to study centromere formation and chromosome organisation. They are successful and effective as gene expression vectors since they remain autonomous and can accommodate larger genes and regulatory regions for long-term expression studies in cells unlike other viral gene delivery vectors currently used. Transferring the essential DNA sequences for HAC formation intact across the cell membrane has been challenging for a number of years. A highly efficient delivery system based on HSV-1 amplicons has been used to target DNA directly to the ES cell nucleus and HAC stably generated in human embryonic stem cells (hESc) at high frequency. HAC were detected using an improved protocol for hESc chromosome harvesting, which consistently produced high-quality metaphase spreads that could routinely detect HAC in hESc. In tumour cells, the input DNA often integrated in the host chromosomes, but in the host ES genome, it remained intact. The hESc containing the HAC formed embryoid bodies, generated teratoma in mice, and differentiated into neuronal cells where the HAC were maintained. The HAC structure and chromatin composition was similar to the endogenous hESc chromosomes. This review will discuss the technological advances in HAC vector delivery using HSV-1 amplicons and the improvements in the identification of de novo HAC in hESc.

## Introduction

De novo human artificial chromosomes (HAC) are small, extrachromosomal elements that contain a functional centromere, enabling their correct replication and segregation as stable normal chromosomes in human cells (Kouprina et al. 2014; Grimes and Monaco 2005), together with the endogenous chromosomes. De novo HAC are generated by introducing specific sequences such as α-satellite (alphoid) DNA into eukaryotic cells. The essential requirement for a functional centromere formation in HAC is α-satellite DNA, containing higher-order repeat sequences and a centromere protein B binding sequence (CENP-B box) (Bergmann et al. 2012).

By mechanisms not yet elucidated, the cell is able to recognise the transgenic DNA as centromeric and seed the deposition of specific protein/epigenetic markers on the exogenous molecules, leading to the formation of a fully active centromere, thus transforming the episomal vector into a full-fledged artificial chromosome (Grimes and Monaco 2005). In human cells, autonomous HAC are characterised by high mitotic stability (Grimes and Monaco 2005; Moralli et al. 2009), unlike other viral vectors which integrate into the host chromosome and may result in insertional mutagenesis (Baum 2007). As there is no limit on the size of the DNA fragments that can be incorporated into HAC, they represent ideal vectors for the delivery of large genomic DNA regions containing genes and their corresponding regulatory elements. HAC containing the entire human genomic hypoxanthine phosphoribosyltransferase (HPRT) region fully complemented the HPRT deficiency in cultured human fibrosarcoma HT1080 cells (Mejía et al. 2001). The de novo HAC structure was relatively simple compared to normal chromosomes (Mejía et al. 2001; Moralli et al. 2009, 2013) and allowed easy characterisation of their composition and chromatin environment.

Known methods for the delivery of large pieces of DNA are inefficient, often resulting in DNA shearing and degradation, which is a major obstacle in developing a HAC expression system in different cell types. Current methods of introducing DNA into cells include the use of lipofecting agents, electroporation and viral and non-viral vectors. Each of these methods shares the problem that only small pieces of DNA can be put into cells without the DNA being damaged. Large DNA fragments have very low transfection efficiencies or break up when entering cells using current methods. This has made it very difficult in the past to get sufficiently large pieces of DNA into cells to form a HAC. In order to use a HAC for gene therapy, the efficiency of delivery of existing methods needs to be improved.

Most gene expression studies in human embryonic stem cells (hESc) utilise lentiviral, adenoviral and adeno-associated (AAV) viral vectors for gene delivery. However, lentiviral vectors integrate randomly at multiple sites within the host genome leading to insertional mutagenesis, and although adenoviral vectors remain episomal, silencing post-transduction may occur. Another disadvantage is that the capacity of AAV and lentiviral vectors is limited to approximately 5 and 10 kb of DNA, respectively.

The herpes simplex virus type 1 (HSV-1)-based amplicon system offers enormous potential as a versatile tool for gene delivery and has several unique features that distinguish it from rest of the viral vector systems and chemical-mediated gene transfer methods. It can accommodate and deliver up to 152 kb of exogenous DNA into the mammalian cells (Saeki et al. 2003; Wade-Martins et al. 2001). Construction of the amplicon DNA vector is simple and flexible. The amplicon DNA does not integrate into the host chromosomes, thus reducing the risk of insertional mutagenesis. The head-to-tail, rolling-circle DNA replication mechanism of HSV-1 also allows amplification of amplicon plasmid as concatemers leading to increased copy number of transgenes and consequently their expression. Most genes in the HSV-1 viral genome are not essential for virus replication and therefore, can be deleted without affecting the virus production from the cultured cells. The lack of viral genes and the availability of amplicon production in a helper virus-free manner results in limited immunogenic and cytotoxic effects. Most importantly, the HSV-1 amplicon can transduce a wide variety of cell types across a wide range of species, including dividing and quiescent cells.

## Generating HSV-1 amplicons

**Fig. 1:**
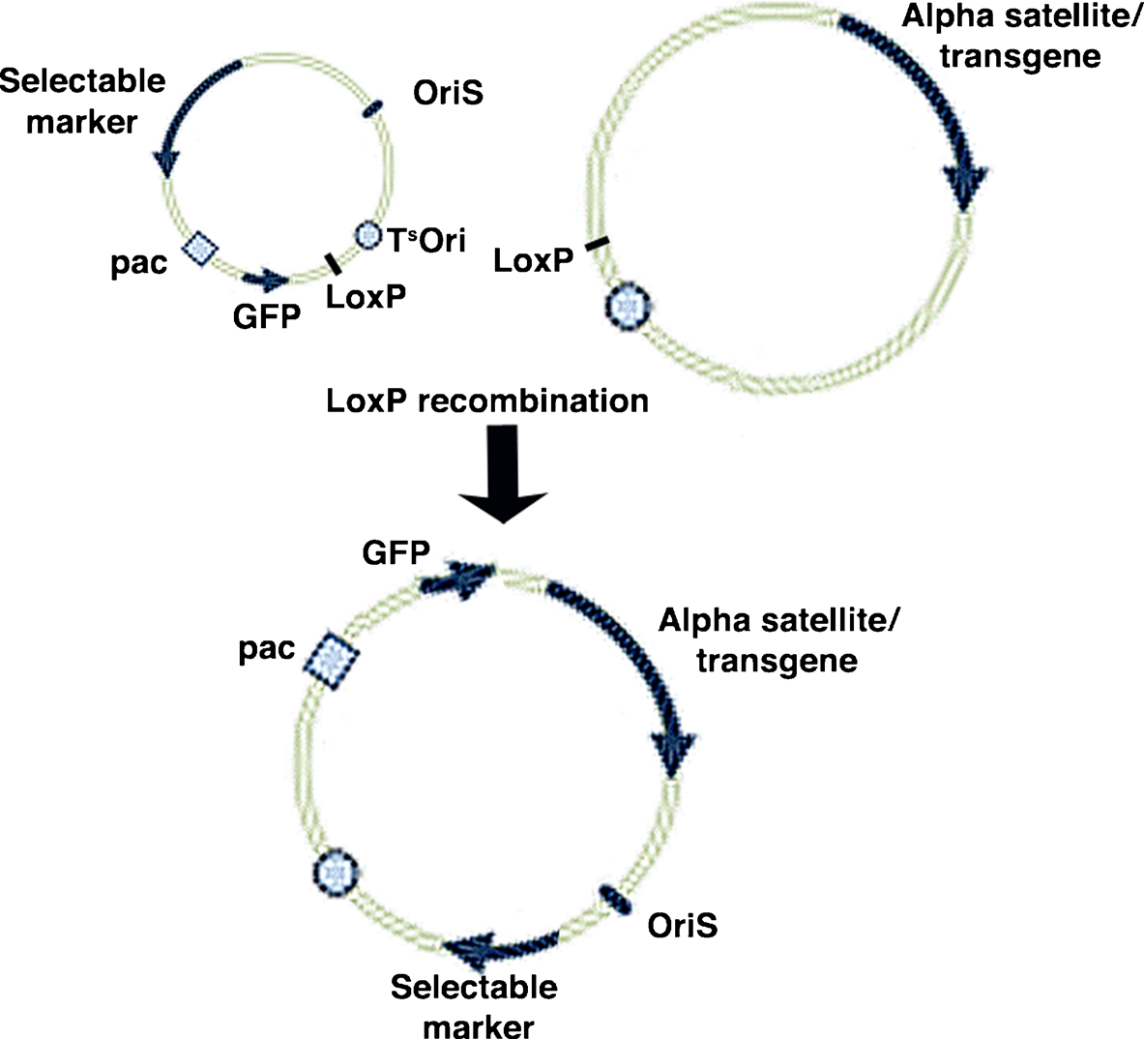
Assembly of HSV-1 amplicon HAC vectors. A large BAC, carrying alpha satellite and/or transgenes, is retrofitted with the HSV-1 necessary elements via LoxP-Cre recombination with a smaller plasmid carrying the HSV-1 origin of replication and packaging signal

**Fig. 2:**
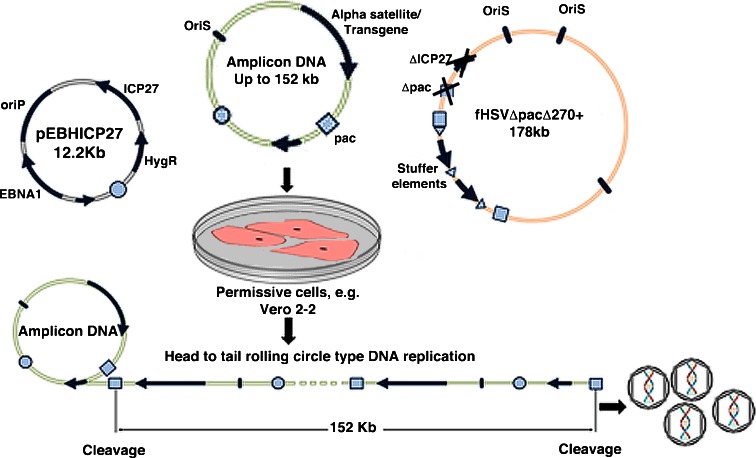
Production of infectious particles. The HSV-1 amplicon HAC vector is transfected into helper cells along with the whole HSV-1 genome (lacking the ICP27 gene and the packaging signal) and a small plasmid expressing the essential HSV-1 protein ICP27. The cell assembles the HSV-1 capsids and packages the HSV-1 amplicon HAC vector

The HAC vectors are firstly retrofitted with the HSV-1 origin of replication and packaging signal (Fig. 1). Infectious particles are then produced by co-transfecting, into the permissive cells such as African green monkey kidney Vero 2-2 cells, (a) the desired amplicon DNA (containing the transgene, the HSV *pac* signal and HSV *oriS*), (b) the fHSVΔpacΔ27 0+ vector containing the complete HSV-1 genome save for the deletion of the *pac* sequence and the essential gene ICP27, and (c) an ICP27 expressing plasmid such as pEBHICP27 (Saeki et al. 2003) (Fig. 2).

Helper functions for the amplification and packaging/cleavage of amplicon DNA into HSV-1 virions are supplied in trans by fHSVΔpacΔ27 0+ and pEBHICP27. However, fHSVΔpacΔ27 0+ itself cannot be packaged because it lacks the packaging signal, the ICP27 gene and HSV-1 origin of replication. Moreover, its size has been increased by the addition of stuffer elements so that the whole construct no longer fits into the HSV virion, thus further reducing the risk of generating replication-competent helper viruses. The HSV-1 amplicon system, therefore, provides a safe and effective mechanism for gene transfer, as amplicons infect the target cells, but lack the components for generating the wild-type virus.

The HSV-1 amplicon DNA can accommodate one or more transgene cassettes as long as its total size does not exceed ∼152 kb. Upon provision of HSV helper functions in trans, the amplicon plasmid undergoes head-to-tail rolling circle replication and results in concatemers of DNA leading to a total size of ∼152 kb which is then cleaved at the *pac* signal and packaged into HSV virions (Fig. 1).

The infectious particles are then used to transduce the target cell. The transgenic material is carried directly to the nucleus, intact (Saeki et al. 2003; Wade-Martins et al. 2001). This feature of the HSV-1 amplicon delivery system is especially important for the generation of human artificial chromosomes and has allowed us to obtain direct HAC formation in human embryonic stem cells for the first time.

## Establishing HAC in human embryonic stem cells

The generation of HAC in hESc and iPS cells is an essential step to gene expression and gene therapy studies. Cells obtained from a patient may be reprogrammed to form iPS cells, then modified ex vivo with a HAC vector containing the desired gene and reintroduced into the patient. hESc could be differentiated into immunoglobulin-producing cells, which could prove useful in vaccine production. The HAC generated in our laboratory and others, have to date been obtained in tumour-derived or immortalised cell lines, such as the human fibrosarcoma cell line HT1080. Before HAC can become fully viable vectors for gene expression studies and potentially gene therapy, it is necessary to study their behaviour in cells that have normal genetic backgrounds such as hESc that can be propagated indefinitely in vitro and can be differentiated in a range of different cell types.

In our study, we assembled HSV-1 amplicon technology HAC input vectors carrying reporter genes (Mandegar et al. 2011). We routinely obtained infectious particles suspensions with titres of 10^8^ amplicon/ml. The vectors were delivered to the hESc cell lines HUES-2 and HUES-10 (Cowan et al. 2004) and the results compared to similar experiments in the human fibrosarcoma HT1080 cells, which are highly efficient at HAC formation (Grimes and Monaco 2005). The transduction efficiency using HSV-1 amplicons is high, ranging from 27 to 40 % in hESc (Mandegar et al. 2011) and up to 70 % in HT1080 cells (Moralli et al. 2006). Following G418 selection, several independent clones can be obtained from each vector in both hESc and HT1080 cells.

## An improved technique for analysing embryonic stem cells by FISH

**Fig. 3:**
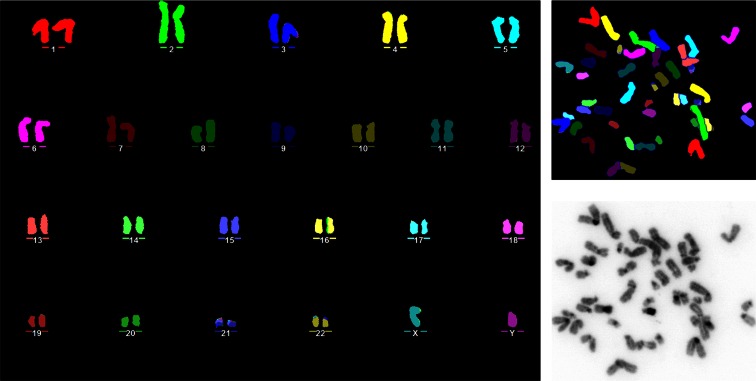
MFISH on HUES10 hESc metaphase spread, obtained with an improved chromosome harvesting procedure

In standard HAC generation experiments, clones are expanded to at least the 10-cm dish stage, before chromosomes are harvested and subjected to fluorescence in situ hybridization (FISH) to determine if the cells contain HACs or integrations. Thus, the karyotypic analysis of a large number of hESc clones can be very time-consuming and expensive. For this reason, the development of an improved harvesting technique, which allows to obtain high-quality metaphase spreads from six-well dishes, proved especially useful for our studies (Moralli et al. 2011). This method relies on overnight incubation with a low dose of nocodazole as synchronizing agent. This increases the mitotic index by tenfold, while maintaining a good chromosome structure. The cells are swelled by treatment in a buffered hypotonic, which dramatically improves chromosome spreading. The quality of the resulting metaphase suspension is such that they can be easily analysed by FISH or MFISH (Fig. 3).

## Characterisation of HAC in hESc

**Fig. 4:**
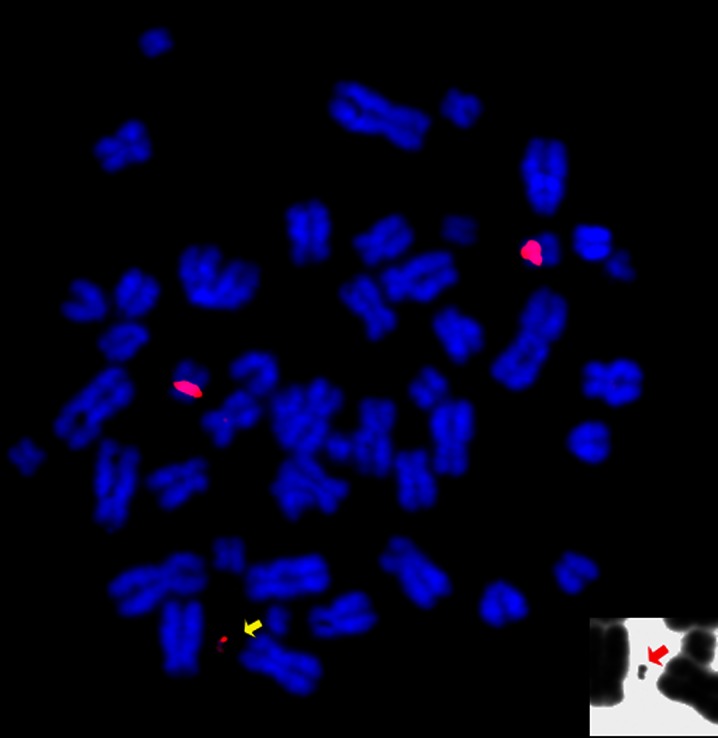
FISH on HAC containing hESc. The metaphase spread has been hybridised with a 17alpha satellite probe (*red signal*) identifying the HAC (*arrow*), along with the endogenous chromosomes 17. The *small inset* show the DAPI staining of the HAC, as a black and white picture

In our studies, hESc clones were analysed by FISH with HAC-specific probes (Fig. 4) to determine if the input vector had integrated randomly into the host cell genome or if had formed a HAC able to segregate independently. While in HT1080, the majority of clones contained integrations and HAC, whereas in the hESc clones, the input DNA had formed HAC in a large percentage of cells and there was no integrated DNA present (Mandegar et al. 2011).

The analysis of the hESc HAC with antibodies against essential centromeric proteins (CENP C) showed that all the HAC had formed a true centromere. When grown in the absence of selection, the HAC were mitotically stable, displaying a daily loss rate of 0.03–0.24 %. Furthermore, immunostaining and RT-PCR analysis of pluripotency markers (Oct4, Sox2, Nanog, TRA-1-60) indicated that the cells derived from the HAC clones remained pluripotent (Mandegar et al. 2011). The pluripotency of the cells was further confirmed following directed differentiation of HAC-containing cells into neuronal type cells and the development of teratomas with the three germ layers following the formation assay. Importantly, the HAC were maintained in the neuronal cells following differentiation, as shown by FISH with specific probes. Although showing some variability, HAC gene expression was sustained over time. Compared to transfection, the HSV-1 amplicon provided highly efficient delivery of small and large DNA segments to HUES-2 cells, and neither transfection nor the HSV-1 amplicon technique was toxic to HUES-2 cells, as shown by measuring the average growth rate post-transduction (Mandegar et al. 2011).

## Conclusion

The HSV-1 amplicon technology is a highly efficient delivery method based on transferring exogenous DNA packaged in HSV-1 amplicons into cells. The amplicons efficiently deliver large DNA containing genes within high-capacity human artificial chromosome vectors into human embryonic stem cells and generate gene expressing HAC in hESc cell lines. The HAC are stable and sustain long-term gene expression. The lack of integrated DNA in hESc following HSV-1 delivery compared to integrated DNA following delivery in cultured cells is a significant difference and will lead to further work on understanding the mechanism of HAC generation in human cells. The differences in genome stability between stem and tumour cells indicate that HAC will be important in gene therapy strategies for monitoring chromosome stability in different cell types. HAC vectors are a viable alternative to gene delivery with viral vectors in hESc with the aim of developing a HAC-based system for the delivery of therapeutic genes regulated with tissue-specific promoters to human target cells for ex vivo treatment. The development of ex vivo strategies for gene therapy of inherited genetic disorders and addressing problems in the areas of cancer, aging and metabolic disorders will be key targets for HAC therapy.

## Abbreviations

AAV: Adeno-associated virus
FISH: Fluorescence in situ hybridization
HAC: Human artificial chromosome
hESc: Human embryonic stem cells
HSV-1: Herpes simplex virus type 1
HPRT: Hypoxanthine phosphoribosyltransferase
